# Isolation and Genetic Characterization of Genotype VII Velogenic Pathotype Newcastle Disease Virus from Commercial Chicken Farms in Central Ethiopia, Distinct from the Local Vaccine Strains

**DOI:** 10.3390/v16081249

**Published:** 2024-08-03

**Authors:** Waktole Yadeta, Elizabeth Amosun, Hawa Mohammed, Wubet Woldemedhin, Kedir Sherefa, Abinet Legesse, Getaw Deresse, Kenaw Birhanu, Takele Abayneh, Belayneh Getachew, Omar Farnós, Amine A. Kamen, Esayas Gelaye

**Affiliations:** 1 School of Veterinary Medicine, College of Agriculture and Veterinary Medicine, Jimma University, Jimma P.O. Box 307, Ethiopia; 2 Pan African University, Life and Earth Sciences Institute, University of Ibadan, Ibadan P.O. Box 200005, Nigeria; 3 Department of Veterinary Microbiology, Faculty of Veterinary Medicine, University of Ibadan, Ibadan P.O. Box 200005, Nigeria; elizabethamosun@yahoo.com; 4 Research and Development Directorate, National Veterinary Institute, Bishoftu P.O. Box 19, Ethiopia; hawa2018mohammed@gmail.com (H.M.); wubyewm@gmail.com (W.W.); kedirsherefa66@gmail.com (K.S.); abinetl781@gmail.com (A.L.); getawderesse2007@gmail.com (G.D.); kenawbr2129@gmail.com (K.B.); takeletefera99@gmail.com (T.A.); belaynehgetachew@gmail.com (B.G.); 5 Viral Vectors and Vaccines Bioprocessing Group, Department of Bioengineering, McGill University, Montreal, QC H3A 0G4, Canada; omar.farnosvillar@mcgill.ca (O.F.); amine.kamen@mcgill.ca (A.A.K.); 6 Food and Agriculture Organization of the United Nations, Sub-Regional Office for Eastern Africa, Addis Ababa P.O. Box 5536, Ethiopia

**Keywords:** Newcastle disease virus, genotype VII, velogenic pathotype, genetic characterization, chicken, Ethiopia

## Abstract

Newcastle disease (ND) is caused by virulent strains of avian paramyxovirus type 1, also known as Newcastle disease virus (NDV). Despite vaccination, the frequency of reported outbreaks in Ethiopia has increased. From January to June 2022, an active outbreak investigation was conducted in six commercial chicken farms across areas of central Ethiopia to identify the circulating NDV strains. Thirty pooled tissue specimens were collected from chickens suspected of being infected with NDV. A questionnaire survey of farm owners and veterinarians was also carried out to collect information on the farms and the outbreak status. NDV was isolated using specific-pathogen-free (SPF)-embryonated chicken eggs and detected using haemagglutination and the reverse transcriptase–polymerase chain reaction (RT–PCR). The genotype and virulence of field NDV isolates were determined using phylogenetic analysis of fusion (F) protein gene sequences and the mean death time (MDT) test in SPF-embryonated chicken eggs. The questionnaire results revealed that ND caused morbidity (23.1%), mortality (16.3%), case fatality (70.8%), and significant economic losses. Eleven of thirty tissue specimens tested positive for NDV using haemagglutination and RT–PCR. The MDT testing and sequence analysis revealed the presence of virulent NDV classified as genotype VII of class II velogenic pathotype and distinct from locally used vaccine strains (genotype II). The amino acid sequences of the current virulent NDV fusion protein cleavage site motif revealed ^112^RRQKR↓F^117^, unlike the locally used avirulent vaccine strains (^112^GRQGR↓L^117^). The epidemiological data, MDT results, cleavage site sequence, and phylogenetic analysis all indicated that the present NDV isolates were virulent. The four NDV sequences were deposited in GenBank with accession numbers F gene (PP726912-15) and M gene (PP726916-19). The genetic difference between avirulent vaccine strains and circulating virulent NDV could explain the low level of protection provided by locally used vaccines. Further studies are needed to better understand the circulating NDV genotypes in different production systems.

## 1. Introduction

Poultry are a good source of animal protein, contributing significantly to food and nutrition security, as well as providing a source of income for a large part of the Ethiopian population [1,2]. The country’s poultry population was estimated to be 41.35 million in 2021/22 [3].

Despite the presence of a large chicken population, the sector’s contribution to the national economy and benefits are relatively limited due to diseases, poor management, the low productivity of local chickens, and market instability [4,5]. Among these constraints, disease is the major challenge affecting the sector [5]. Newcastle disease is one of the most important poultry diseases, causing substantial economic losses for the country due to its high morbidity and mortality [6].

Newcastle disease (ND) is caused by virulent strains of avian paramyxovirus type 1 (APMV-1), also known as Newcastle disease virus (NDV), of the genus Orthoavulavirus belonging to the subfamily Avulavirinae, family Paramyxoviridae [7,8,9,10]. The NDV genome is a single-stranded negative-sense RNA with six transcriptional units (3′-N-P-M-F-HN-L-5′). The F protein cleavage site sequence plays an important role in NDV virulence [8,9]. It has a wide host range of more than 250 domestic bird species, including chickens, turkeys, pheasants, guinea fowl, uscovy ducks, geese, and free-ranging wild birds, including migratory waterfowl, shorebirds, passerines, and wild pheasants [10].

The ND virus strains are classified into two classes based on the amino acid identity of the L protein and the genome size. Class I carries strains of NDV with large genomes (15,198 nt), which are mostly avirulent for chickens, and class II strains have shorter genomes, which carry lentogenic, mesogenic, and velogenic strains of NDV. Class II viruses are then classified into 22 genotypes [7,10,11].

The first cases of ND were discovered in Java, Indonesia, and Newcastle-upon-Tyne, England, in 1926. Since then, the disease has primarily spread in Central and South America, Africa, and Asia [6]. In Ethiopia, the first confirmed case of the disease was reported in 1971, following an outbreak in Asmara city. Later, the disease gradually spread throughout the country [2,12].

According to [13], Ethiopia reported an average of 50 outbreaks per year to the World Organisation for Animal Health between 2005 to 2015. This raised concerns regarding the efficacy of existing vaccination efforts, and whether the apparent outbreaks occurred due to incompatibility between field and vaccine strains, ineffective vaccination procedures, or the generation of novel genotypes under high immune pressure. The generation of novel genotypes may lead to outbreaks, since most of the currently used vaccine strains (mostly derived from genotypes I and II) offer inadequate protection against other genotypes [14]. This has been highlighted by reports of genotype VI and VII in vaccinated chickens [15,16]. The fusion protein cleavage site (Fcs) of NDV contributes significantly to virulence and membrane fusion. Previous studies found that changing phenylalanine (F) to lysine (L) at position 117 of the virulent strain fusion protein, which has the polybasic amino acid fusion protein cleavage site (Fcs) motif ^112^RRQKR↓F^117^, induces substantial syncytium formation in the infected cells [17].

In general, current genetic information of the circulating Newcastle disease virus genotypes is important in diagnosis, vaccine development, vaccination strategies, and establishing a control strategy. Hence, the goal of this study was to isolate and characterize Newcastle disease virus from outbreaks in commercial poultry farms in three districts of central Ethiopia.

## 2. Materials and Methods

### 2.1. Study Area

The study was conducted from January to June 2022 in purposively selected intensive commercial poultry farms with suspected NDV outbreaks in central Ethiopia. Accordingly, pathological samples were obtained from clinically diseased chickens from Bishoftu (Ada’a district) and Mojo (Lume district) towns of the East Shewa Zone and Addis Ababa city, which are potential sources of poultry production in the country.

**Figure 1:**
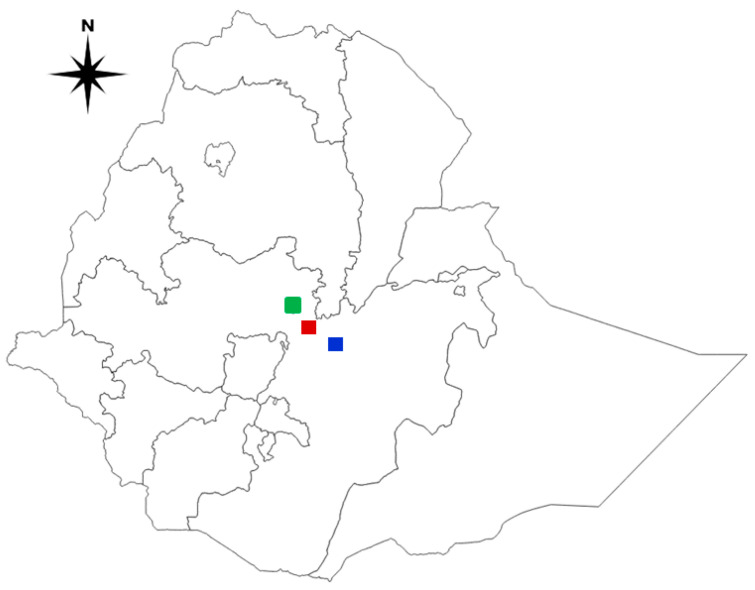
A map of Ethiopia depicts the study areas where suspected Newcastle disease outbreak samples were collected. Green: Addis Ababa; Red: Bishoftu; Blue: Mojo. The map was created with QGIS software version 3.10.0-A.

Bishoftu town is located in the East Shewa zone of the Oromia region in the central highlands of Ethiopia at 8°45′ N and 38°59′ E. The town is located in the warm-to-cool sub-moist mid-highland at an altitude of approximately 1920 m above sea level (masl) (Figure 1). It is located approximately 45 km southeast of the capital, Addis Ababa. The average minimum and maximum temperatures range from 10.9 to 27.0 °C, with a mean value of 18.9 °C. The town has an average annual rainfall of 686.9 mm and an average relative humidity of 60.0% [18].

Mojo town is located in the East Shewa zone of the Oromia region, Ethiopia, 66 km southeast of Addis Ababa and lies at 8°35’ N and 39°7’ E at an altitude of 1790 masl. The area gains rainfall twice a year, which is known as the long and short rainy season. The main rainy season extends from June to September. The average annual rainfall, temperature, and mean relative humidity are 776 mm, 19.4 °C and 59.9%, respectively [18]. There is no official statistical report of poultry populations in either Bishoftu or Mojo towns, but, according to [3], the poultry population of the East Shewa zone, which includes Bishoftu, Mojo, and other towns not included in this study, is estimated at 859,616 [3].

Addis Ababa is the capital and largest city of Ethiopia. It is known to have a daily average temperature ranging from 9.9 to 24.6 °C, with a mean annual rainfall of 1224 mm and an elevation of 2110 masl [18]. There is no official statistical report of the poultry population in Addis Ababa city.

### 2.2. Study Population

Active outbreaks were investigated on six private poultry farms with 500 to 10,000 chickens per farm in the study area. The study animals were chickens of different ages, breeds, sexes, and origins that showed clinical signs of NDV infection. Information on the occurrence of ND outbreaks was acquired by establishing good communication with the corresponding farm owners, veterinarians, district animal health service departments, and production managers through physical meetings, social media, and phone calls.

### 2.3. Study Design

An active disease outbreak investigation was carried out from January to June 2022 in intensive poultry farms with suspected ND outbreaks. The affected farms were small-, medium-, and large-scale poultry farms in Bishoftu and Mojo towns of the East Shewa zone and Kality subcity of Addis Ababa city in Central Ethiopia.

### 2.4. Sample Size Determination and Sampling

Since the objectives of the study were to isolate ND virus from clinical samples and perform a molecular characterization of the virus strains currently circulating in the study area, the focus was towards isolating the virus causing the outbreak instead of an epidemiological study, which needed a formula-based sample size determination. Moreover, due to a limited resource for sample processing and to increase the chance of isolating the exact virus strain causing the outbreak, the study area, farms, and sampled chickens were purposively selected based on the report of an active outbreak, accessibility, and the willingness of the farm owners to allow sample collection and the presence of active clinical signs of ND. In such studies, [10] recommends the collection of five samples per affected farm. Such a method was employed in the works of [16,19,20,21]. Thus, from all six farms investigated, 30 chickens showing clinical signs of ND were sampled. From each sampled chicken, at least two to three pathological tissue samples were collected based on the clinical signs seen (neurological or digestive) and pathological findings. In total, 85 tissue samples were collected.

### 2.5. Data Collection

#### Questionnaire Survey

A semi-structured questionnaire was developed to collect useful information for the study, and farm owners or veterinarians working on each farm were asked for their willingness to participate. Before the interview, all six participants (one from each farm) gave their verbal consent.

The questionnaire for the interview included questions about the farm name, location, date of the outbreak, production type, breed, age, sex, flock size, management system, origin of the birds, vaccination history (name of vaccines used, company name and country of production, batch number if available, and vaccination program), clinical signs, morbidity, mortality, and associated economic impacts. The format used to document the outbreak investigations is available in Supplementary File S1.

### 2.6. Clinical and Postmortem Examination

A general inspection and examination of diseased chickens were used to assess the clinical signs the birds were showing in a flock and individually. A postmortem examination of each chicken was performed at the postmortem room of the National Veterinary Institute (NVI) of Ethiopia to identify the necropsy findings and aseptically collect the needed pathological samples for the study.

### 2.7. Sample Collection, Transportation, and Storage

Thirty chickens with typical clinical signs of NDV infection were collected and taken to the postmortem facility of the NVI. The chickens were killed through cervical dislocation and a standard postmortem examination was conducted to collect the required samples. Samples from the lungs, intestine, spleen, caecal tonsils, brain, liver, and heart were aseptically collected based on the clinical signs and postmortem findings observed in each chicken. The collected samples were placed in sterile isotonic phosphate-buffered saline (PBS), pH 7.2, with penicillin (2000 units/mL), streptomycin (2 mg/mL), and gentamycin (50 μg/mL). The samples were immediately transferred to the virology laboratory of the institute and kept at −80 °C until processing [10].

### 2.8. Isolation and Molecular Detection of ND Virus

The frozen tissue samples stored at −80 °C were thawed at room temperature and processed for virus isolation as indicated in the WOAH Terrestrial Manual [10]. Approximately 1 g of pooled samples was minced into small pieces using sterile scissors and ground with a sterile mortar and pestle in a class II biosafety cabinet. Tissue suspensions (10% *w*/*v*) were prepared by adding 9 mL of PBS containing antibiotics and antifungals.

The suspension was transferred to a 10 mL glass test tube and the supernatant fluids were obtained through clarification using centrifugation at 5000× *g* for 10 min at +4 °C. One millilitre of supernatant fluid was harvested and aliquoted into a sterile cryovial tube for use in the isolation of the virus [10].

The isolation of the virus was performed through the inoculation of 0.2 mL supernatant fluid of tissue suspension into the allantoic cavity of 11-day-old embryonated specific-pathogen-free (SPF) chicken eggs (three eggs per sample) according to the standard virus isolation methods described previously [10,22]. The SPF chicken eggs were obtained from VALO BioMedia, Germany. After inoculation, the eggs were incubated at 35–37 °C for 2–5 days. The inoculated eggs were candled in a dark room every 24 h to check embryo viability. Each egg containing a dead embryo on each day and all eggs at the end of the incubation period were chilled at 4 °C. Allantoic fluids were harvested and tested using the haemagglutination (HA) test for their ability to haemagglutinate chicken red blood cells. Three serial passages in embryonated eggs were performed for each sample to increase the virus titre.

The haemagglutination (HA) test was conducted in the serology laboratory of NVI as a screening test. Both rapid haemagglutination and micro-haemagglutination tests using a V-bottom microwell plate (Nunc, Roskilde, Denmark) were used to determine the presence of a haemagglutinating agent in the allantoic fluid, as outlined by the World Organization for Animal Health [10]. A micro-haemagglutination test using a V-bottom microwell plate was also used to quantify the amount of Newcastle disease virus in a suspension. The assay was performed by carrying out twofold serial dilutions of the allantoic fluid in a microwell plate and then testing to determine an end point. The test result was used to determine the amount of hemagglutinin in the suspension and expressed as an HA unit. The last well that showed complete agglutination was the well that contained one HA unit. After the HA unit was determined, the HA titre was calculated as the reciprocal of the dilution that produced one HA unit [10].

All HA-positive allantoic fluids were analysed using the conventional reverse transcription polymerase chain reaction (RT–PCR) test at the molecular biology laboratory of NVI. RNA was extracted from allantoic fluids using the RNeasy^®^ Mini kit (Qiagen, Hilden, Germany) following the manufacturer’s instructions. Master mix was prepared using the Qiagen One-Step RT–PCR kit. The RT–PCR mixture of each sample consisted of 5 µL of RNase-free water, 5 μL of kit-supplied buffer (5×), 5 µL of kit-supplied Q-solution, 2 µL of forward primer M610-5′-CTGTACAATCTTGCGCTCAATGTC-3′ (5 pM), 2 µL of reverse primer NCDVF-581-5′-CTGCCACTGCTAAGTTGTGATAATCC-3′ (5 pM) (Eurofins Genomics, Vienna, Austria), 1 μL of kit-supplied enzyme mix, 1 μL of kit-supplied deoxynucleotide triphosphates (10 mM), and 4 µL of the sample (RNA), resulting in a final reaction volume of 25 μL [23].

The amplification was performed using a thermal cycler (2720, Applied BioSystems, Waltham, MA, USA) with a PCR protocol of cDNA synthesis at 50 °C for 30 min, an initial denaturation at 95 °C for 15 min, followed by 35 cycles of denaturation at 94 °C, annealing at 53 °C, extension at 72 °C, each for 30 s, and a final extension at 72 °C for 10 min. The amplified PCR products were analysed with 1.5% agarose gel (Cleaver Scientific Ltd., Rugby, UK, lot no: 14160704) and the gel was stained with 5 µL of an intercalating dye (20× Pronasafe, Condalab^®^, Madrid, Spain, Cat. No: 4687). Briefly, 10 μL of each PCR product was mixed with 5 μL of 5× gel loading dye (Hi Media^®^, Mumbai, India) and loaded into separate wells. Six microliters of Gel Pilot^®^ DNA molecular marker starting at 100 bp (Qiagen, Hilden, Germany, lot no: 25.1) was run simultaneously in the first and last wells to estimate the size of the sample PCR product at 120 V electric current for 1 h on an electrophoresis apparatus (EC 2060, Holbrook, NY, USA). Illumination with ultraviolet light (UVITEC, Cambridge, UK) was used to visualize stained DNA. A positive result for NDV showed a band size of approximately 1100 bp.

### 2.9. Sequencing and Phylogenetic Analysis

One positive allantoic fluid from each farm was further processed for sequencing. RNA was extracted from allantoic fluids using an RNeasy^®^ Mini kit (Qiagen, Hilden, Germany) following the manufacturer’s instructions. The master mix was prepared with the QIAGEN One-Step RT–PCR kit [15,24]. The RT–PCR mixture of each sample consisted of 10 µL of RNase-free water, 10 μL of kit-supplied buffer (5×), 10 µL of kit-supplied Q-solution, 4 µL of forward primer AK-gene 4-Fow 5′-TGAAAACGACGGCCAGTAGATGATRACAACATGTAGRTG-3′ (5 pM), and 4 µL of reverse primer AK-gene 4-Rev 5′-CAGGAAACAGTATGATGACCGGCTAACYGCRCGGTCCAT-3′ (5 pM), 2 μL of kit-supplied enzyme mix, 2 μL of kit-supplied deoxynucleotide triphosphates (10 mM), and 8 µL of the sample (RNA), resulting in a final reaction volume of 50 μL. The amplification was performed using a thermal cycler (Applied Biosystems 2720 Thermal Cycler, Singapore) with a PCR protocol of 50 °C for 30 min, an initial denaturation at 95°C for 15 min, followed by 35 cycles of denaturation at 94 °C, annealing at 60 °C, extension at 72 °C for 30 s, and a final extension at 72 °C for 10 min.

From 50 µL of the amplified PCR products of each sample, 5 µL was used for the analysis in gel electrophoresis with 1.5% agarose gel. The remaining 45 µL of each PCR product was used for a further sequencing process. A positive result for the F-gene was identified by the presence of a band at approximately 704 bp. Then, the positive PCR product (45 µL) was purified using the Wizard^®^ SV Gel and PCR Clean-Up System (Promega, Walldorf, Germany) following the manufacturer’s instructions. The concentration of the purified PCR product was quantified using a microvolume spectrophotometer (NanoDrop 2000c, Thermo Scientific™, Newington, NH, USA). The concentration of the quantified purified PCR product was adjusted following the requirements set by the sequencing company. The purified PCR products were mixed with the sequencing primers and submitted to a commercial sequencing company (LGC Genomics, Berlin, Germany). Raw sequence data obtained from the company were analysed using Staden Package software (https://staden.sourceforge.net/, accessed on 16 October 2021) [25] and the fragments were assembled using Vector NTI Advance™ 11.5 software (Invitrogen, Carlsbad, CA, USA). The sequences were aligned using BioEdit 7.1.3.0 [26]. The F protein sequences were analysed to determine the presence of mutant cleavage sites.

The degree of sequence similarity search was conducted using the BLAST program (NCBI). All sequence analyses were conducted in MEGA7 [27]. Multiple sequence alignments were performed using Clustal W. Phylogenetic relationships among the current isolates and isolates from other geographical areas were determined based on phylogenetic trees constructed using the neighbour-joining algorithm.

### 2.10. Pathogenicity Assessment

To assess the virulence of the viruses, a mean death time (MDT) test was performed on all PCR-positive allantoic fluids following previously described methods [10,28,29,30]. For the test, allantoic fluid was diluted 10-fold in PBS (pH 7.2) and inoculated into 11-day-old SPF-embryonated chicken eggs. The eggs were candled two times a day (morning and afternoon) for embryonic death and the results were recorded. The NDVs in allantoic fluids causing embryonic death (MDT) of up to 60 h, from 61 to 90 h, and more than 90 h were designated velogenic, mesogenic, and lentogenic, respectively [10].

### 2.11. Data Management and Statistical Analysis

The collected data were recorded on Microsoft Excel 2013 (Microsoft Corp., Redmond, WA, USA) spreadsheets. The Statistical Package for Social Sciences (Ibm SPSS Statistics version 20) was used for the descriptive statistical analysis. Morbidity, mortality, and case fatality rates were estimated in accordance with different variable categories.

## 3. Results

### 3.1. Questionnaire Survey

Along with the sample collection, the questionnaire survey was distributed to collect different parameters of the farms and animals studied. A total of six farms with different characteristics were investigated (Table 1). The suspected ND outbreaks were reported in the months of March, April, and May 2022 during the entire study period. Rose 308 and Cobb 500 were the broiler breeds, while Lohman brown and Bovans brown were layer breeds reared on the investigated farms. The Sasso breed was reared for dual purposes (broilers and layers). All recorded outbreaks occurred in chicks aged between 25 and 62 days old.

During the outbreaks, 6700 chickens were affected by ND (23.1% of all the chickens on the ND positive farms) and 4742 (16.3%) died. The disease was observed on five of the six farms visited. Morbidity was highest at farms three and four in Mojo (50%), followed by farm five (31%) in Addis Ababa, and farm two (14.5%) and farm one (13%) in Bishoftu. The highest mortality rate was observed in farm four at Mojo (42.5%), followed by farm five at Addis Ababa (26.6%), farm three at Mojo (27.1%), farm two (10%), and farm one (7%) at Bishoftu.

The case fatality rate was the highest at Farm four in Mojo (85%), followed by farm five (84.2%) at Addis Ababa and farm two (68.9%) at Bishoftu, farm three (54.2%) at Mojo, and farm one (53.8%) in Bishoftu (Table 2).

The survey recorded information on vaccination history on each farm. Accordingly, Cevac^®^ New L, Ceva Santé Animale, imported from France, Nobilis^®^ MA5 + Clone 30, Intervet International B.V., imported from the Netherlands, and live Newcastle HB1/Lasota, produced at NVI in Ethiopia, were the ND vaccines commonly used on farms. Although there was a minor variation in the schedule of ND vaccination, all the farms followed the companies’ recommendations.

The survey also enabled an estimation of the direct economic losses resulting from chickens dying from ND. Direct economic losses were estimated from the weighted average price of each animal that died. Thus, the average cost of a single 4- to 5-week-old chicken dying from ND was calculated to be ETB 100, which was equivalent to USD 1.85 (USD 1 = ETB 54). From the five active ND outbreaks that occurred, the direct economic loss from the deaths of 4742 chickens was, therefore, estimated as ETB 474,200 (USD 8781.5).

### 3.2. Clinical and Postmortem Findings

**Figure 2:**
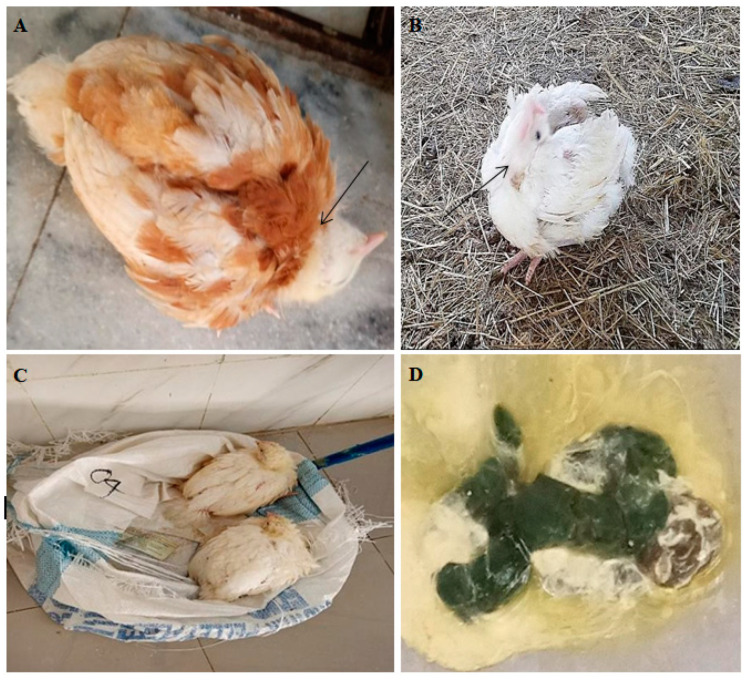
Chicken pictures taken during clinical examination: (**A**,**B**) torticollis (arrow), (**C**) depression, and (**D**) greenish diarrhoea.

Affected chickens underwent clinical and postmortem examinations. Table 3 shows the typical clinical signs observed: twisted necks (torticollis), circling, ocular discharge, ruffled feathers, and respiratory distress with gasping, depression, and greenish diarrhoea (Figure 2).

**Figure 3:**
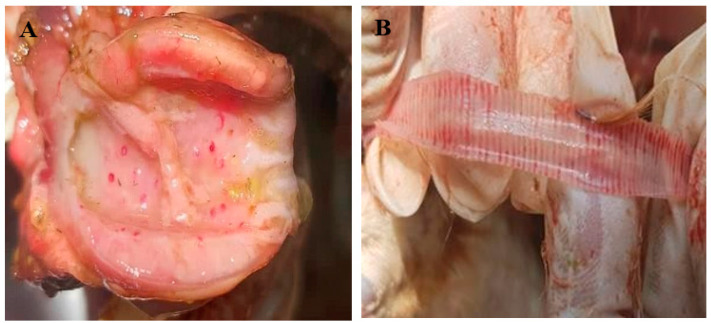
Postmortem findings of chickens infected with ND virus. (**A**) Haemorrhage in the proventriculus and (**B**) haemorrhage in the trachea.

The postmortem examination revealed that more than 80% of the birds had ND-related gross lesions, such as haemorrhage in the trachea, proventriculus, and ceca. The lesions were mostly seen in birds with digestive system symptoms (Figure 3).

### 3.3. Isolation of ND Virus

Pooled samples from each of the 30 ND-suspected chickens were inoculated into 11-day-old embryonated chicken SPF eggs with four replications per sample. One negative control (without a sample) with four replications was also incubated. A total of 17 samples showed embryonic death starting from 48 h of the first passage, while one sample showed embryonic death in the second passage. The negative control eggs showed no embryonic death (Table 4).

### 3.4. Haemagglutination Test

A haemagglutination test was used to screen the harvested allantoic fluids. In total, 31 allantoic fluids (four replications per sample were pooled into one) were tested using a rapid slide and microtiter plate HA test, and 11 were found to be positive in both tests. Using a microtiter plate HA test, the HA unit was determined with results ranging from 1/2 to 1/1024. The HA titre, which is the reciprocal of the HA unit, ranged from 16 to 1024 (Table 4).

### 3.5. Molecular Detection of ND Field Viruses

**Figure 4:**
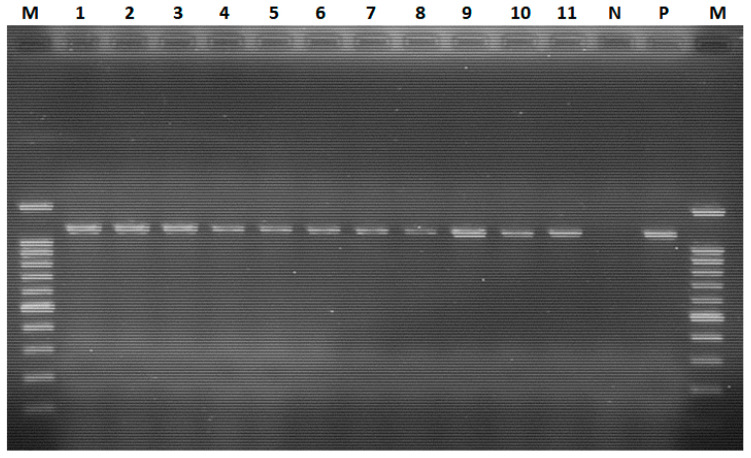
Conventional RT–PCR-based detection of the ND virus. Lane M: 100 bp plus molecular ladder; lanes 1–3: positive samples from farm 1; lane 4: positive sample from farm 2; lanes 5 and 6: positive samples from farm 3; lane 7: positive sample from farm 4; lanes 8–11: positive samples from farm 5; lane N: negative control; lane P: positive control.

The conventional RT–PCR assay detected the presence of ND virus in the harvested allantoic fluid with an amplicon of 1100 bp. The findings showed that all 11 allantoic fluids tested positive using haemagglutination, and rapid slide agglutination tests were also positive for ND virus using RT–PCR (Figure 4).

### 3.6. Sequence Analysis of ND Field Virus Isolates

**Figure 5:**
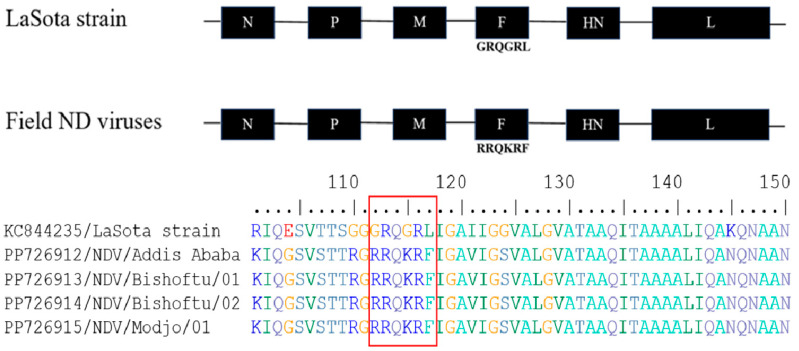
Newcastle disease virus genome with the fusion (F) protein gene cleavage site sequence of LaSota vaccine strain showed a ^112^GRQGRL^117^ sequence, whereas the current ND virus field isolates revealed a ^112^RRQKRF^117^ sequence motif. Amino acid sequence alignment of the F gene from the LaSota vaccine strain and Ethiopian field NDV isolates with the six amino acid sequence signature or motif at the cleavage site are highlighted in the box.

The current four NDV field isolates from each outbreak area were sequenced and deposited in GenBank with accession numbers for the fusion (F) protein gene (PP726912-PP726915) and matrix (M) protein gene (PP726916-PP726919). Only four sequences were clean and had adequate nucleotide coverage among the samples collected from the five farms, therefore, they were included for a further amino acid sequence analysis. The sequence GRQGRL was found at the cleavage site of the F protein gene in the avirulent LaSota vaccine strain, whereas the RRQKRF sequence motif was found in the field isolates (Figure 5), indicating that the current field NDV isolates were virulent strains.

### 3.7. Phylogenetic Analysis of ND Viruses

**Figure 6:**
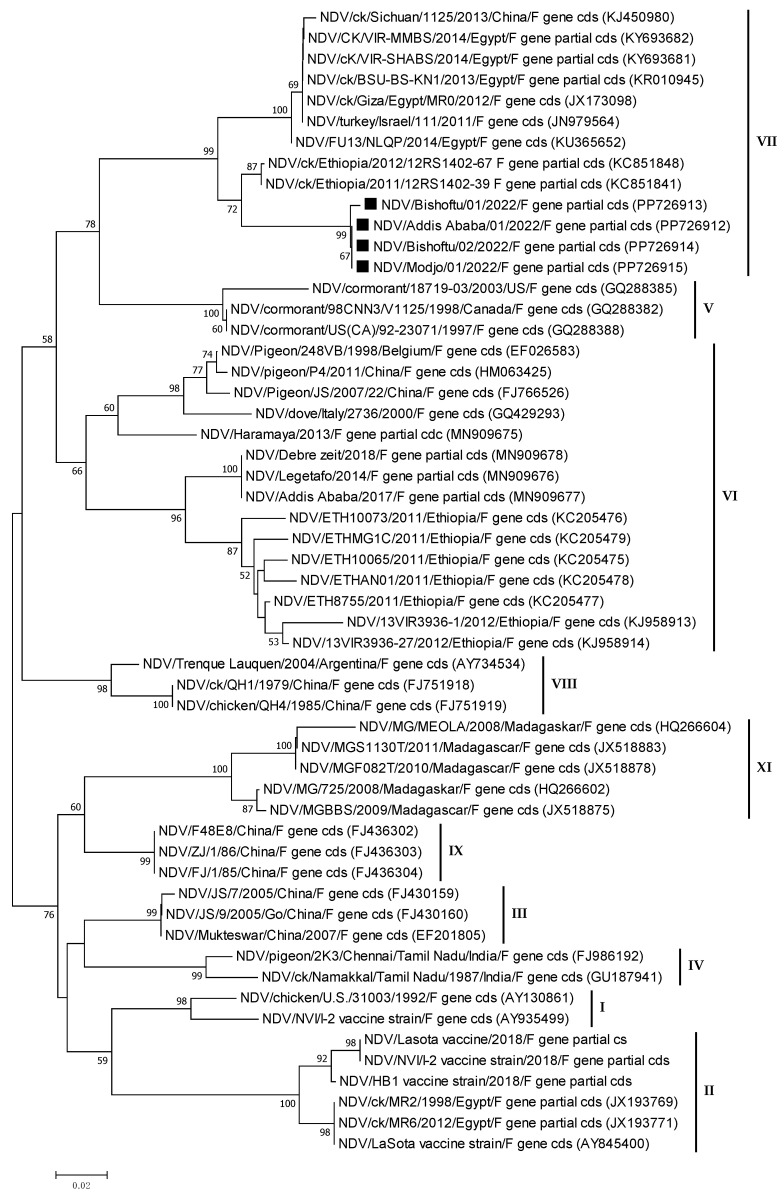
Phylogenetic tree of Newcastle disease viruses based on the partial nucleotide sequences (563 bp) of the Fusion protein gene (F gene). The phylogenetic tree was constructed using 55 NDV nucleotide sequences, which included four field isolates from the current investigation (PP726912-15, labelled with a rectangle), vaccine strains, previously sequenced Ethiopian isolates, and homologous sequences retrieved from GenBank. MEGA7 software (https://www.megasoftware.net/, accessed on 16 October 2021) was used to compute the neighbour-joining method with the maximum composite likelihood nucleotide substitution model and the pairwise deletion option. Percentage bootstrap scores greater than 50% (out of 1000 replicates) are displayed next to each branch. The current four isolates belonged to genotype VII of class II ND viruses.

The phylogenetic analysis was carried out on nucleotide sequences from the M and F genes. The tree was built using the outbreak NDV isolates from the current investigation and homologous sequences of additional NDV sequences retrieved from GenBank. The phylogenetic analysis of partial F gene sequences (563 bp) revealed that all isolates from the study areas were genotype VII (Figure 6). The genetic mean distance within the four Ethiopian NDV field isolates was o.oo1. The current field NDV isolates were phylogenetically more closely related to the previously sequenced Ethiopian virulent two NDV isolates (KC851841 and KC851848), which were genotype VII lineage 5.

### 3.8. Pathogenicity of ND Virus Filed Isolates

All eleven NDV-positive allantoic fluids evaluated using the mean death time (MDT) assay exhibited an MDT of less than 60 h (41–57 h) (Table 4). This suggests that all ND virus field isolates had a velogenic pathotype.

## 4. Discussion

In this study, an active outbreak investigation was conducted on six farms, with ND identified in five of them. The outbreak on these farms resulted in overall morbidity, mortality, and case fatality rates of 23.1%, 16.3%, and 70.8%, respectively. This finding was higher than that of a previous study in Ethiopia, which revealed overall morbidity, mortality, and case fatality rates of 18.7%, 9.5%, and 50.5%, respectively [31]. However, the observed mortality rate was lower than that reported in Chad (55%) [32], in Eritrea (97.7%) [33], and in India (21.1%) [34]. This variation may be associated with the chickens’ immunity status and age, concurrent diseases, and strain of the virus.

Despite routine vaccination with vaccines from three different companies (Cevac^®^ New L, Ceva Santé Animale, France; Nobilis^®^ MA5 + Clone 30, Intervet International B.V., the Netherlands; and live Newcastle HB1/Lasota, NVI, Ethiopia) produced from genotype I and II, outbreaks continued to occur in the study areas. This was consistent with previous reports of ND in vaccinated chicken flocks [35,36,37]. This could be associated with genetic and antigenic differences between the vaccine strains and circulating field strains [15,38,39]. However, poor vaccine application or vaccine quality could not be ruled out, necessitating more investigations to determine whether the outbreak was the result of vaccine failure.

ND creates significant economic losses due to its high morbidity and mortality [13,40]. Although this study lacked a clear and measurable economic data analysis, the findings indicated that chicken mortality caused by ND were directly associated with significant economic losses.

This study documented clinical signs of NDV infection, such as torticollis, circling, ocular discharge, ruffled feathers, legs paralysis, respiratory distress with gasping, depression, and greenish diarrhoea. This was consistent with studies in Eritrea [33] and India [34]. This study identified that respiratory distress was more common in broilers. This could be related to broilers’ high metabolic rate (fast growth), which requires more oxygen transport to cells.

The majority of the chickens had gross pathological lesions consistent with velogenic viscerotropic ND. The lesions were characterized by pin-point haemorrhages in the proventriculus and generalized haemorrhage in the caecal tonsils and trachea. This was consistent with previous findings [31,34].

The current investigation found that almost all PCR-positive samples resulted in embryonic mortality (50–100%) within 42 h (2 days) of post-inoculation into 11-day-old embryonated chicken SPF eggs. This could be explained by the high virulence nature of the virus strain. This finding was in agreement with previous reports [41,42], which indicated that all chicken embryos died within two days of inoculation.

The harvested allantoic fluids were screened using a haemagglutination test. The study indicated that all HA-positive samples with HA titres of 1:16–1:1024 tested positive for ND using RT–PCR. This finding was consistent with the results of a study by Osman et al. [43].

In this investigation, the molecular characterization of NDV field isolates based on nucleotide sequences of the matrix (M) and fusion (F) genes revealed that all isolates from the study areas belonged to genotype VII of class II viruses. This suggested that the same NDV genotype circulated throughout the poultry farms, independent of their geographical location. This was consistent with previous findings on genotype VII lineage five circulated in northwest and central Ethiopian villages and live bird marketplaces [15,44]. The findings highlighted the risk of the spread of NDV genotype VII strains in Ethiopian commercial chicken farms and household rural chickens, with one possible explanation being the use of genotype-mismatched vaccines (genotype II). The presence of identical strains in these farms showed that the virulent virus strain is widely distributed in the country. However, a further investigation is required because the number of field isolates in this study was quite limited. The occurrence of genotype VII in this study differed from other findings [16,45,46] that reported the circulation of genotype VI in small-scale poultry farms in different commercial chicken farms in central and eastern Ethiopia. This suggests the presence of different NDV genotypes in the country.

The pathogenicity testing of the NDV field isolates in this investigation revealed that all isolates were virulent, with an MDT of less than 60 h. Although this pathogenicity result should be confirmed through the in vivo assessment of virus virulence using the intra-cerebral pathogenicity index (ICPI) test, which is considered the most sensitive and widely used test for measuring virus virulence, the findings were consistent with previous ones revealing the circulation of virulent NDV in Ethiopian chickens [15,16,44].

The NDV strains with F residue at position 117 of the fusion gene had a larger tissue tropism resulting in a more virulent disease. RRQRR↓F is a virulent virus constituting of residue F at position 117 (F117) of the F protein, whereas the F gene amino acid sequence of the LaSota vaccine strain, an avirulent virus, revealed the presence of the GRQGR↓L motif at the fusion protein cleavage site (L117). The virulent NDV virus normally possesses residue F at position 117, which is necessary for membrane fusion [9,17,47,48]. The current field NDV isolates showed F (phenylalanine) at position 117, whereas the vaccine virus (LaSota strain) showed L (leucine), indicating that the field viruses were pathogenic while the vaccine strain had low virulence. This finding was consistent with the WOAH Terrestrial Manual, which states that most APMV-1 viruses that are pathogenic for chickens have the sequence ^112^R/K-R-Q/K/R-K/R-R^116^ at the C-terminus of the F2 protein and F (phenylalanine) at residue 117, the N-terminus of the F1 protein, whereas viruses with low virulence have sequences in the same region of ^112^G/E-K/R-Q-G/ER^116^ and L (leucine) at residue 117 [10].

The epidemiological data recorded during the outbreak investigation, the MDT findings, the Fusion protein cleavage site sequence motifs, and the phylogenetic analysis all supported that the current NDV isolates were virulent strains.

## 5. Conclusions

ND is one of the most prevalent poultry diseases in Ethiopia. This study revealed that outbreaks of ND in commercial chicken farms in three districts of central Ethiopia resulted in significant economic losses, regardless of whether ND vaccination was performed using locally produced or imported vaccines. The current outbreak investigation revealed high mortality, morbidity, and case fatality rates. The molecular characterization and mean death time of NDV field isolates causing outbreaks in the studied farms revealed the circulation of virulent NDV strains belonging to genotype VII of class II viruses, which were distinct from the vaccine strains used (genotype II). This suggests that the current vaccines used in the study areas were incompatible, resulting in inadequate protection. Therefore, when producing vaccines and designing vaccination programs, it is recommended that the genotype of the circulating strains be considered. To fully understand the underlying causes of outbreaks, it is also necessary to investigate the efficacy of currently available ND vaccines as well as potential risk factors. Given the small number of clinical samples and viral isolates considered in this study, further research is needed to better understand the molecular epidemiology of NDV and identify the circulating NDV genotypes throughout Ethiopia’s different chicken production systems.

## Figures and Tables

**Table 1:** Characteristics of the poultry farms and animals studied.

Farm #	Age (Days)	Breed	Production Type	Flock Size	Location
1	31	Rose 308	Broilers	10,000	Bishoftu
2	35	Lohman Brown	Layers	10,000	Bishoftu
3	25	Cobb 500	Broilers	2000	Mojo
4	29	Bovans brown	Layers	4000	Mojo
5	28	Sasso	Dual purpose	3000	Addis Ababa
6	62	Sasso	Dual purpose	500	Bishoftu

**Table 2:** Morbidity, mortality, and case fatality rates recorded in Newcastle disease outbreaks in three study areas in central Ethiopia.

Farm #	Location	No. of Susceptible Chicken	Morbidity (%)	Mortality (%)	Case Fatality Rate (%)
1	Bishoftu	10,000	1300 (13%)	700 (7%)	53.8%
2	Bishoftu	10,000	1450 (14.5%)	1000 (10%)	68.9%
3	Mojo	2000	1000 (50%)	542 (27.1%)	54.2%
4	Mojo	4000	2000 (50%)	1700 (42.5%)	85.0%
5	Addis Ababa	3000	950 (31%)	800 (26.6%)	84.2%
6	Bishoftu	500	None	None	None
	Total	29,000	6700 (23.1%)	4742 (16.3%)	70.8%

**Table 3:** Clinical signs observed in Newcastle-disease-affected chickens.

Farm #	Breed	Production Type	Clinical Signs Observed
1	Rose 308	Broilers	Respiratory distress with gasping, depression, paralysis of the legs, and greenish diarrhoea
2	Lohman Brown	Layers	Torticollis, circling, and depression
3	Cobb 500	Broilers	Respiratory distress with gasping, depression, greenish diarrhoea, and torticollis
4	Bovans brown	Layers	Ocular discharge, greenish diarrhoea, and depression
5	Sasso	Dual purpose	Depression, greenish diarrhoea, and ocular discharge
6	Sasso	Dual purpose	Depression, torticollis, and greenish diarrhoea

**Table 4:** Results of ND virus isolation, time of embryo death, haemagglutination, and PCR tests.

Farm Code	Sample Code	No of Eggs Died	Mortality (%)	Time of Embryo Death (hr)	HA Test Result	HA Titre	PCR Result	Mean Death Time (hr)
Farm 1	1	3, P1	75	48 (2), 72 (1)	Pos	128	Pos	42
	2	3, P1	75	48 (2), 72 (1)	Pos	128	Pos	51.4
	3	4, P1	100	48 (2), 72 (2)	Pos	512	Pos	45
	4	0	0	-	Neg	Neg	-	
	5	1, P3	25	96	Neg	Neg	-	
Farm 2	6	4, P1	100	48 (3), 72 (1)	Pos	1024	Pos	41
	7	2, P1	50	96 (1), 96 (1)	Neg	Neg	-	
	8	0	0	-	Neg	Neg	-	
	9	1, P3	25	72 (1)	Neg	Neg	-	
	10	0	0	-	Neg	Neg	-	
Farm 3	11	0	0	-	Neg	Neg	-	
	12	4, P1	100	48 (2), 72 (2)	Pos	512	Pos	57
	13	3, P1	75	48 (2), 72 (1)	Pos	128	Pos	49
	14	1, P3	25	96 (1)	Neg	Neg	-	
	15	0	0	-	Neg	Neg	-	
Farm 4	16	1, P2	50	48 (2)	Neg	Neg	-	
	17	0	0	-	Neg	Neg	-	
	18	0	0	-	Neg	Neg	-	
	19	1, P3	25	96 (1)	Neg	Neg	-	
	20	4, P1	100	48 (3), 72 (1)	Pos	1024	Pos	43
Farm 5	26	3, P1	75	48 (2), 72 (1)	Pos	512	Pos	56
	27	2, P2	50	48 (1), 72 (1)	Pos	16	Pos	48
	28	3, P1	75	48 (1), 72 (2)	Pos	64	Pos	52
	29	2, P2	50	48 (1), 96 (1)	Pos	16	Pos	42
	30	1, P3	25	96 (1)	Neg	Neg	-	
Farm 6	21	0	0	-	Neg	Neg	-	
	22	0	0	-	Neg	Neg	-	
	23	1, P1	25	96 (1)	Neg	Neg	-	
	24	0	0	-	Neg	Neg	-	
	25	0	0	-	Neg	Neg	-	
Neg control	31	0	0	-	Neg	-	-	No death

hr, hour; P, passage number; Neg, negative; Pos, positive. Figures in parentheses represent the number of dead embryos.

## Data Availability

The datasets generated and/or analysed during the current study are available in the manuscript and the Supplementary Materials. Raw data and additional photos can be made available from the corresponding authors upon request.

## Supplementary Materials

The following supporting information can be downloaded at: https://www.mdpi.com/article/10.3390/v16081249/s1. The sequence information obtained in this study can be retrieved from the NCBI website using the accession numbers (PP726912-PP726919). The format used to record the outbreak investigation and laboratory analysis findings can be found in Supplementary File S1.
